# Plasma Circulating Nucleic Acids Levels Increase According to the Morbidity of *Plasmodium vivax* Malaria

**DOI:** 10.1371/journal.pone.0019842

**Published:** 2011-05-17

**Authors:** Bernardo S. Franklin, Barbara L. F. Vitorino, Helena C. Coelho, Armando Menezes-Neto, Marina L. S. Santos, Fernanda M. F. Campos, Cristiana F. Brito, Cor J. Fontes, Marcus V. Lacerda, Luzia H. Carvalho

**Affiliations:** 1 Laboratório de Malária, Centro de Pesquisa René, Fundação Oswaldo Cruz, Belo Horizonte, Minas Gerais, Brazil; 2 Gerência de Malária, Fundação de Medicina Tropical Dr. Heitor Vieira Dourado, Manaus, Amazonas, Brazil; 3 Departamento de Clínica Médica, Universidade Federal de Mato Grosso, Cuiaba, Mato Grosso, Brazil; State University of Campinas, Brazil

## Abstract

**Background:**

Given the increasing evidence of *Plasmodium vivax* infections associated with severe and fatal disease, the identification of sensitive and reliable markers for vivax severity is crucial to improve patient care. Circulating nucleic acids (CNAs) have been increasingly recognized as powerful diagnostic and prognostic tools for various inflammatory diseases and tumors as their plasma concentrations increase according to malignancy. Given the marked inflammatory status of *P. vivax* infection, we investigated here the usefulness of CNAs as biomarkers for malaria morbidity.

**Methods and Findings:**

CNAs levels in plasma from twenty-one acute *P. vivax* malaria patients from the Brazilian Amazon and 14 malaria non-exposed healthy donors were quantified by two different methodologies: amplification of the human telomerase reverse transcriptase (hTERT) genomic sequence by quantitative real time PCR (qPCR), and the fluorometric dsDNA quantification by Pico Green. CNAs levels were significantly increased in plasma from *P. vivax* patients as compared to healthy donors (*p<0.0001*). Importantly, plasma CNAs levels were strongly associated with vivax morbidity (*p<0.0001*), including a drop in platelet counts (*p = 0.0021*). These findings were further sustained when we assessed CNAS levels in plasma samples from 14 additional *P. vivax* patients of a different endemic area in Brazil, in which CNAS levels strongly correlated with thrombocytopenia (*p = 0.0072*). We further show that plasma CNAs levels decrease and reach physiological levels after antimalarial treatment. Although we found both host and parasite specific genomic sequences circulating in plasma, only host CNAs clearly reflected the clinical spectrum of *P. vivax* malaria.

**Conclusions:**

Here, we provide the first evidence of increased plasma CNAs levels in malaria patients and reveal their potential as sensitive biomarkers for vivax malaria morbidity.

## Introduction

*Plasmodium vivax* malaria threatens almost 40% of the world's population, with an upper estimate of 300 million cases each year [1]. Fortunately, after a long time being neglected under the contemptible designation of benign infection, vivax malaria has gained increasing attention in recent years.

In the last decade, a series of case reports and longitudinal studies carried out in India [2], [3], Papua in Indonesia [4], [5], Papua New Guinea [6] and Brazil [7] have demonstrated association of *P. vivax* infections with severe or even fatal outcomes, with incidence and morbidity rates similar to those for *P. falciparum*. Consequently, costs due to hospitalization have significantly raised as well as the need for intensive care, which helped vivax malaria to be placed in a higher status of public health emergency [7].

Compared to falciparum malaria, there are remarkably large knowledge gaps in the pathophysiology of vivax malaria, and the true spectrum of clinical disease in endemic areas remains unknown [8]. The few studies that have addressed the pathogenesis of vivax malaria showed that the different clinical presentations of vivax malaria might be related to the intensity of pro-inflammatory responses [9], [10], [11], [12]. Inflammatory cytokines such as TNF-alpha and antioxidant agents have been associated with clinical severity of *P. vivax* infections [13], [14]. Nevertheless, data validating their sensitivity and reliability as predictors of severe disease are scarce. Consequently, the identification of highly sensitive biomarkers for malaria vivax morbidity is crucial to prevent life threatening complications.

Most of the DNA and RNA in the human body are located within cells, but small physiologic amounts of nucleic acids can also be found circulating freely in the blood. These DNA, RNA, and small RNA molecules may arise from both: i) active release of nucleic acids from living cells, or ii) break down of dying cells that release their contents into the blood. The term Circulating Nucleic Acids (CNAs) refers to cell free segments of DNA or RNA found in the bloodstream. Their existence in human plasma was first reported more than 60 years ago [15], however, no interest was shown in the presence of DNA in the circulatory system until high DNA levels were demonstrated in the blood of patients with cancer [16]. Elevated plasma CNAs levels have now been detected during other acute illnesses and injuries. Examples include lupus erythematosus [17], [18], diabetes [19], trauma [20], stroke [21], and myocardial infarction [22], [23]. Furthermore, high usefulness of CNAs levels in the diagnosis of infections in febrile patients and as a prognostic marker in septic patients has been shown [24]. Their applications in clinical diagnosis and prognosis have continuously grown and further studies on CNAs showed that these nucleic acids could be a powerful non-invasive approach to a wide range of clinical disorders [25].

Aiming at finding sensitive and reliable biomarkers for *P. vivax*, herein we tested the usefulness of plasma CNAs levels as markers for the morbidity of vivax malaria. We investigated the CNAs levels in plasma from *P. vivax* infected patients with different clinical presentations and found significant higher levels of CNAs in *P. vivax* infected patients, as compared to age-matched healthy donors. We found that plasma CNAs levels were closely correlated with variations in body temperature, platelets counts, and increased in a linear fashion with the clinical spectrum of vivax malaria, evaluated here by scoring patients' clinical and hematological parameters.

## Results

**Figure 1:**
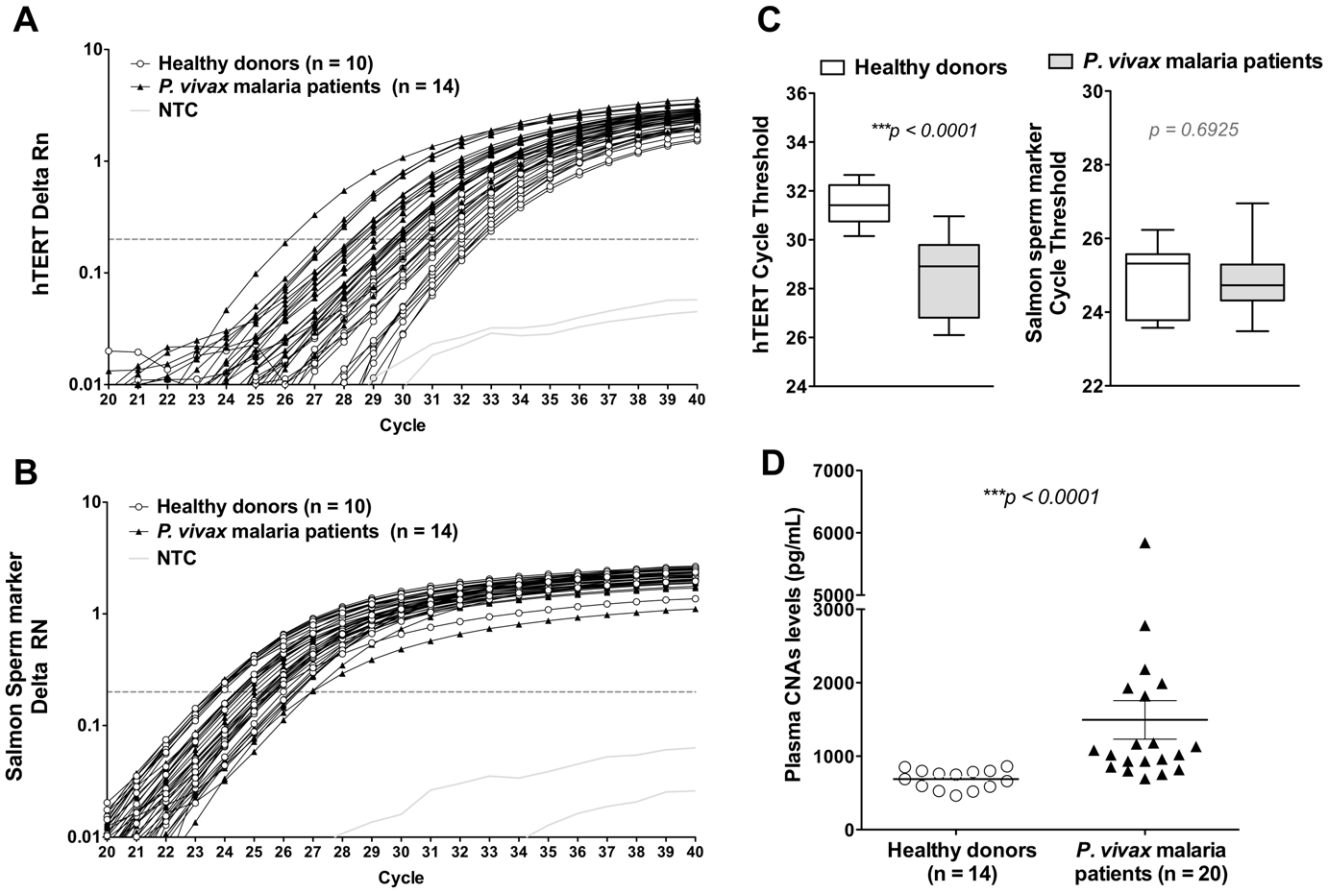
Increased CNAs levels in plasma from *P. vivax* patients. CNAs levels were quantified in plasma from acute *P. vivax* patients or healthy donors by measuring the amplification of the hTERT human genomic sequence (A) as compared to the amplification of the *O. keta* Y chromosome marker (B) for the salmon sperm DNA spiked into plasma samples before CNAs purification. (C) Comparison of the mean cycle threshold (Ct) from the hTERT or the *O. keta* Y chromosome marker in CNAs samples purified from *P. vivax* patients or non-exposed healthy donors. (D) Fluorometric dsDNA quantification of CNAs levels in plasma by the Quant-iT™ Pico Green methodology. Statistical analyses were performed using the Mann-Whitney test. A p value<0.05 was considered significant.

CNAs levels were measured in plasma from *P. vivax* patients by qPCR amplification of the genomic sequence of the human single copy gene hTERT and by fluorometric quantification of the dsDNA content with the Quant-iT™ Pico Green Reagent. The amplification plot of hTERT shows that the mean cycle threshold (Ct) achieved in CNAs samples from *P. vivax* patients (mean Ct 28.6±1.5) was significantly lower than the one reached in samples from healthy donors (mean Ct 31.5±0.79) (*p<0.0001*) (Fig. 1A and 1C). As the amount of DNA theoretically doubles every cycle during the exponential phase of qPCR, these results suggest that the levels of this target sequence in the CNAs preparation from *P. vivax* patients are at least 8-fold higher than in healthy donors. In fact, a difference of 11,6× between the hTERT levels in plasma from *P. vivax* patients (1.278 pg/ml) and healthy donors (0.1098 pg/ml) was confirmed when a standard curve, built from a serial dilution of an amplified sample of hTERT sequence, was used to interpolate the hTERT concentrations in the samples (Figure S1). To normalize the amount of nucleic acids purified and inputted in qPCR experiments, 5 ng of salmon sperm DNA was spiked into plasma samples before CNAs purification (Fig. 1B). As expected, the specific sequence of salmon sperm DNA was similarly amplified in *P. vivax* patients and healthy donors plasmas (Fig. 1B and 1C, *p = 0.6925*).

The increased levels of total CNAs in plasma from *P. vivax* patients were confirmed by quantification of dsDNA with Quant-iT™ Pico Green Reagent (Fig. 1D) (1494.7±1169.7 in vivax patient vs. 689.03±131.54 pg/ml in healthy donors, *p<0.0001*).

**Figure 2:**
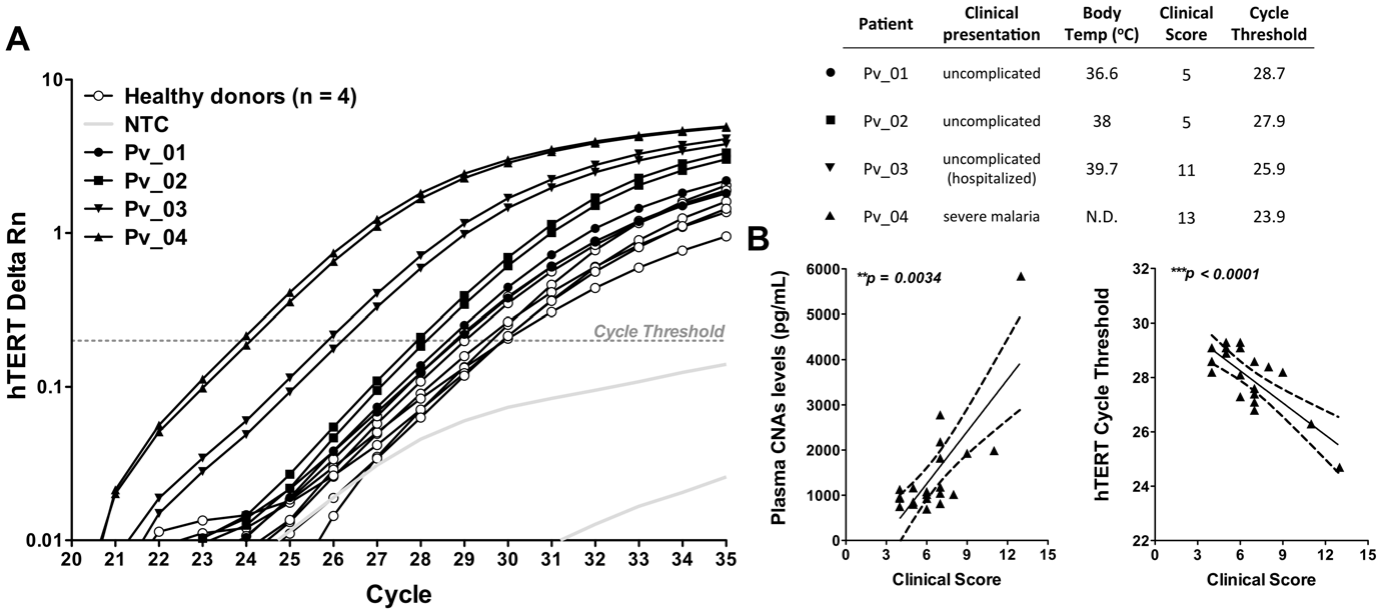
Plasma CNAs levels reliably correlate with the *P. vivax* clinical spectrum. CNAs levels were quantified in plasma from *P. vivax* patients with different clinical presentations. (A) Amplification of the genomic sequence of hTERT by qPCR in four healthy controls, and *P. vivax* patients (Pv_01 to 04) scored according to clinical/hematological parameters. Only four patients are shown for illustration purposes. (B) Correlation between the final clinical score of *P. vivax* patients (n = 21) and their plasma CNAs levels (pg/ml) (Spearman r = 0.6092, *p = 0.0034*), or their Ct for the amplification of the hTERT (Pearson r = −0.7897, *p<0.0001*).

To investigate the potential of CNAs as biomarkers for malaria morbidity, we compared the levels of CNAs in plasma from patients with different clinical presentations, and scored according to clinical and hematological parameters (Table S1). Figure 2A illustrates the qPCR amplification of the hTERT genomic sequence in plasma from four *P. vivax* patients and four unexposed-controls. Sensitive changes in hTERT amplification were observed according to the slightest increase in the clinical score. Furthermore, significantly higher levels of CNAs were found in plasma isolated from patients who presented fever at the time of blood collection (febrile patients) compared to plasma samples from non-febrile patients, as revealed by the two different methodologies: amplification of hTERT genomic sequence by qPCR (*p = 0.0376*) and the quantification of dsDNA content with Quant-iT™ Pico Green (*p = 0.0023*) (data not shown).

To confirm whether CNAs levels reflect disease morbidity, the sum of scores attributed to each patient (Table S1) was plotted against the CNAs levels detected in plasma with the Quant-iT™ Pico Green or the mean cycle threshold detected by qPCR amplification of the hTERT genomic sequence (Fig. 2B). A clear correlation (Spearman *r = 0.4795*, *p = 0.0034*) was found between the CNAs levels and the intensity of clinical malaria. These data were confirmed when the Cts from the amplification of hTERT were analyzed (Pearson *r = −0.7111*, *p<0.0001*) (Fig. 2B).

**Figure 3:**
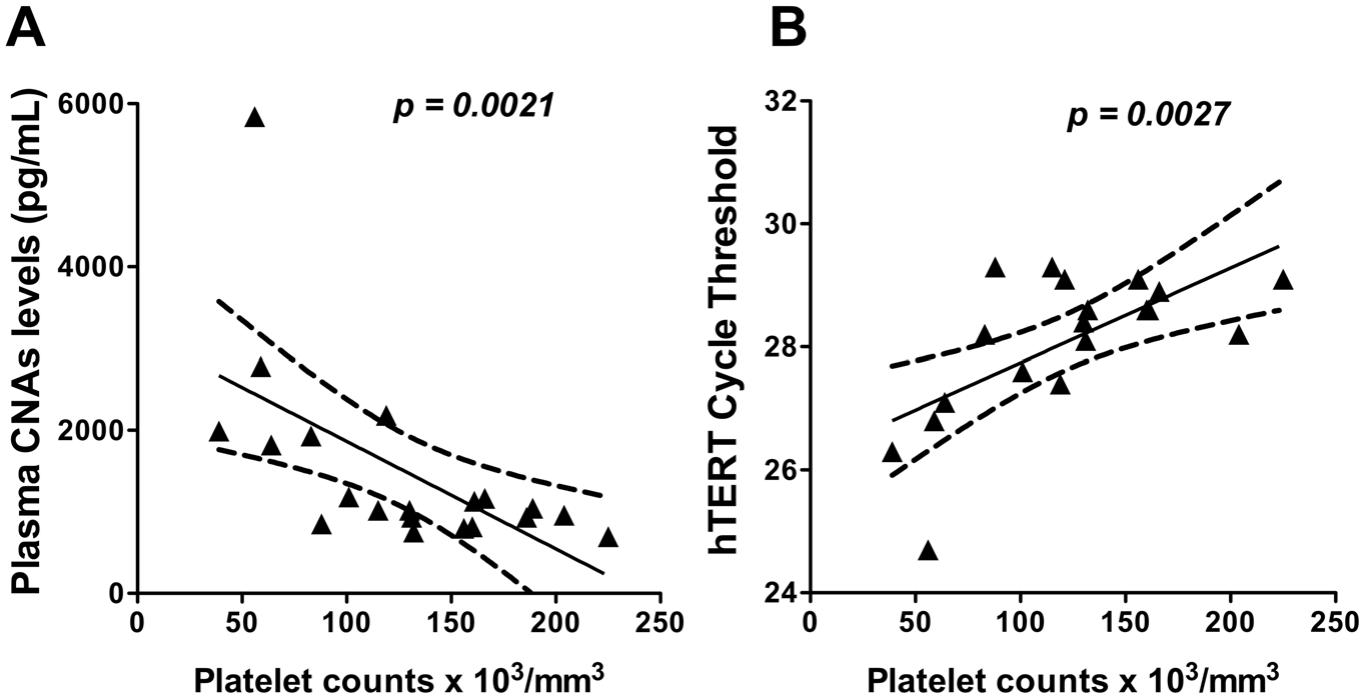
Correlation between plasma CNAs levels and malaria vivax thrombocytopenia. Correlation of plasma CNAs levels with platelet counts in symptomatic vivax malaria patients. The dsDNA levels measured by Pico Green (A) and the mean cycle threshold for hTERT amplification (B) were plotted against the platelet counts. Spearman (r = −0.6451) and Person (r = 0.6479) correlations were used respectively in A and B. A p value<0.05 was considered significant.

Platelet activation exerts thrombotic and pro-inflammatory functions and their unbalanced activation contributes to life-threatening outcomes in diseases such as heart attack, stroke, and cancer [26]. Both platelets [27] and platelet derived microparticles (PMPs) [10] have been associated with clinical manifestations of malaria. We thus investigated if plasma CNAs levels may be associated with thrombocytopenia and/or others hematological parameters, such as WBC and RBC counts, hemoglobin and hematocrit levels, mean corpuscular hemoglobin (MHC) and mean platelet volume (MPV). Among all parameters investigated, we found a strong negative correlation between CNAs levels, assessed by dsDNA quantification with Pico Green, and platelet counts (spearman *r = −0.6451*, *p = 0.0021*) (Fig. 3A). These findings were confirmed when the mean Ct obtained after qPCR amplification of the genomic sequence for hTERT gene was plotted against platelet levels (Pearson *r = 0.6479*, *p = 0.0027*) (Fig. 3B).

To further confirm the association between CNAS levels and *P. vivax* morbidity, we assessed the CNAS levels in plasma from an additional group of *P. vivax* patients whose selection was carried-out in a different hospital of the Amazon area, Cuiaba, MT (∼1500 miles from Manaus, AM). Once the clinical protocol used at the hospital in Cuiaba was different from Manaus (FMT-HVD), we were unable to build a similar clinical score. For this reason, we compared the CNAS levels in these samples with thrombocytopenia, a common hematological disturbance seen in malaria morbidity in the Amazon area [28]. By analyzing the amplification of hTERT, it was possible to demonstrate a significant correlation (Pearson r = 0.745, *p = 0.0072*) between CNAS levels and thrombocytopenia in *P. vivax* patients from Cuiaba (Figure S2A).

As this study provides the first description of circulating nucleic acids in malaria infection, we evaluate CNAs levels in a small group of *P. falciparum* patients who sought for care at Cuiaba's hospital (n = 9). CNAs levels were significantly higher in samples from falciparum malaria patients as compared to healthy donors (*p = 0.038*; not shown). Importantly, CNAs levels in patient's plasma clearly correlated with thrombocytopenia (Figure S3A) and the occurrence of fever during acute *P. falciparum* infection (Figure S3B).

**Figure 4:**
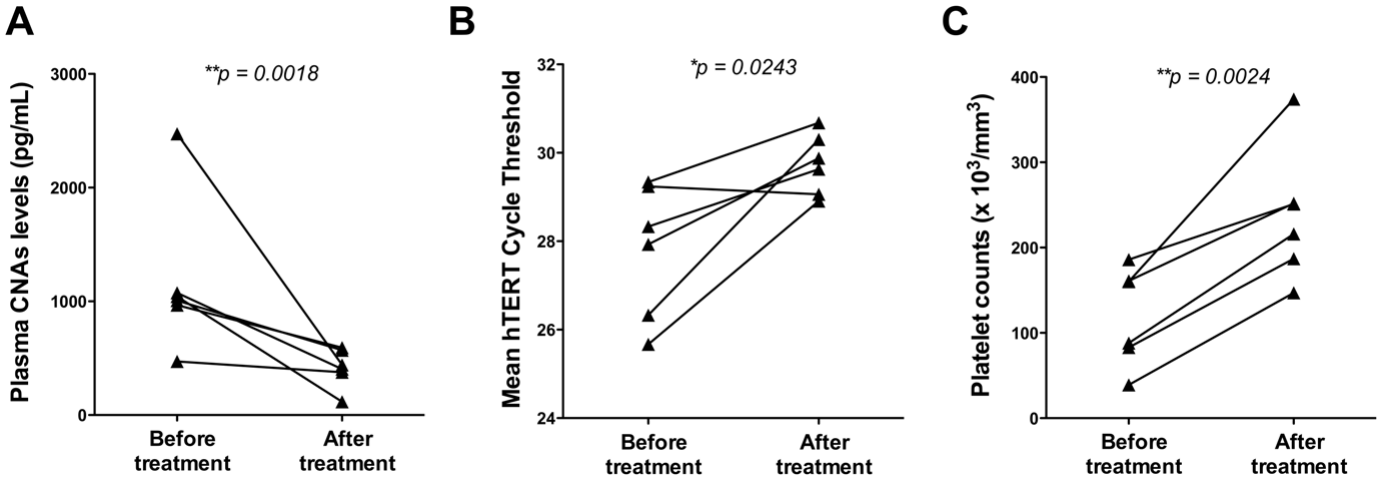
Plasma CNAs levels decrease after anti-malarial chemotherapy. For 6 patients who showed up during convalescence, the CNAs levels in plasma were assessed by (A) fluorescence quantification of dsDNA with the Pico Green methodology or (B) comparison of the mean cycle threshold for the qPCR amplification of hTERT genomic sequence. (C) platelet counts measured during admission and convalescence. Statistics were performed as follow: Mann-Whitney test for panel A, and two tailed t test for panels b and C. A p value<0.05 was considered significant.

In six patients attended at the FMT-HVD (Manaus, AM), the CNAs levels were further assessed 7 days after antimalarial chemotherapy. As shown in Fig. 4A, CNAs levels decreased after specific treatment (*p = 0.0428*). The comparison of the mean Ct obtained after qPCR amplification of the hTERT in plasma samples from acute vs. treated patients confirmed these findings (*p = 0.0243*) (Fig. 4B). Seven days post-treatment, the platelet counts returned to physiological levels (Fig. 4C). These data were further confirmed in patients from Cuiaba area (n = 10) (Figure S2B). In those samples, CNAs levels were significantly diminished after 7–10 days of chemotherapy.

**Figure 5:**
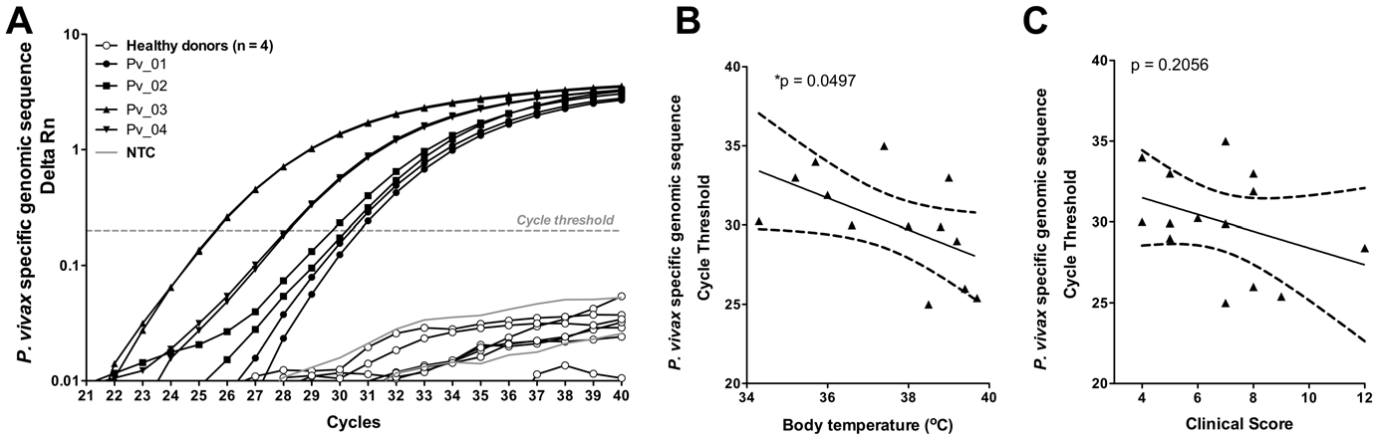
*Plasmodium* specific genomic sequences circulating in plasma from *P. vivax* patients. The presence and levels of *P. vivax* specific plasma CNAs were investigated in samples from *P. vivax* patients with different clinical presentations by qPCR amplification of a specific *P. vivax* genomic sequence. (A) For illustration purpose, qPCR amplification of a *P. vivax* specific genomic sequence in four healthy controls, and four *P. vivax* patients (Pv_01 to 04) scored according to clinical/hematological parameters is shown. (B) Pearson correlation between the Ct of parasite specific genomic sequence and the body temperature measured at the time of blood collection (*r = −0.5535*, *p = 0.0497*) or the clinical score of the patients (*r = −0.3604*, *p = 0.2056*). A p value<0.05 was considered significant.

It is reasonable to speculate that parasite specific DNA is present among the CNAs circulating in plasma. To confirm this, we assessed the levels of *P. vivax* derived-CNAs in plasma in an attempt to investigate their use as a streamline diagnostic and prognostic tool. For this purpose, specific primers were designed to amplify a genomic sequence unique to *P. vivax*. As expected, amplification of this genomic sequence was not detected in samples from healthy donors (Fig. 5A). Furthermore, although parasite specific CNAs levels were weakly associated with the presence of fever at the time of blood sampling (Ct vs. body temperature, *r = −0.5535*, *p = 0.0497*) (Fig. 5B), they were not associated with the clinical spectrum of the disease (*r = −0.3604*, *p = 0.2056*) (Fig. 5C). Also, parasite specific CNAs genomic sequences did not reflect peripheral parasitemia (*r = −0.3735*, *p = 0.1884*).

## Discussion

This study is the first to investigate the use of plasma levels of cell-free circulating nucleic acids (CNAs) as a marker of *P. vivax* malaria morbidity. We show here that CNAs levels in plasma from *P. vivax* patients increase linearly with the clinical spectrum of the disease. This confirms that this powerful marker can also be used in malaria as a sensitive indicator of inflammation and injury. In fact, plasma CNAs levels have been regarded as a noninvasive universal cancer biomarker [29] as their levels have been shown to be distinctly increased in most patients with solid tumors (E.g. lung [30], colon [31], cervical [32], ovarian [33], breast [34], testis [35], bladder [36], and prostate [37]), allowing their discrimination from patients with nonmalignant disease or healthy individuals. Plasma CNAs levels have also been associated with the severity of several other inflammatory disorders [17], [18], [19], [20], [21], [22], [23].

Other molecules circulating in plasma, such as adhesion molecules [38], pro-inflammatory cytokines [39], the superoxide dismutase-1 [14], and, more recently, microparticles [10], have been suggested as biomarkers for human *P. vivax* malaria as their levels are often associated with malaria clinical manifestations. Nevertheless, we believe that CNAs offer a more sensitive tool since qPCR amplification of hTERT, a specific single copy human genomic sequence, revealed that levels as low as 100 fentogram of CNAs could be detected circulating in plasma, and were able to discriminate different degrees of disease morbidity (Figure S1).

We show here that plasma CNAs reach physiologic levels after 7–10 days of antimalarial chemotherapy and patient's recovery. It has been shown that clearance of cell-free DNA from the bloodstream occurs rapidly; the half-life time of fetal DNA in the blood of mothers after delivery was approximately 16 minutes [40]. Cell-free DNA seems to be eliminated by different manners including renal and hepatic mechanisms as well as degradation by plasma nucleases [29]. It is unknown whether a different clearance time is also contributing to the higher levels of cell-free DNA in malaria patients. The kinetics by which CNAs levels rise and fall during acute malaria requires further investigation.

The source of CNAs levels during malaria remains unknown. Apoptosis and necrosis have been pointed as the main source of cell-free DNA circulating in blood [41], [42]. Usually apoptosis-induced cleavage of DNA results in DNA fragments of approximately 180 bp; thus, quantification of a small and a long PCR product allows indirect inferences about the underlying cell-death entity. Although apoptosis has not been directly addressed in this study, our results do not rule out this possibility, as most of the fragments amplified were in the range of 90 bp to be suitable for qPCR analysis. In malaria, apoptosis is a process highly represented in the annotation of gene expression profile of acute infection as revealed by several microarray studies involving both human and mouse models [43], [44]. Nevertheless, it was recently shown that apoptosis and or necrosis might not be the main sources of CNAs in plasma of patients with a variety of other conditions, and active release of free circulating DNA by living cells was pointed as a plausible mechanism [45]. At this time, it is unknown whether apoptosis and/or DNA release contribute to the higher levels of cell-free DNA observed here in *P. vivax* patients.

Thrombocytopenia (platelet counts <150,000/mm^3^) is a common hematological finding in patients with *Plasmodium* infection particularly in vivax malaria [28], [46]. Recent studies carried out in northwest India highlighted the higher occurrence of severe thrombocytopenia in *P. vivax* in comparison to either *P. falciparum* monoinfection or mixed infections [47], [48]. We show here that CNAs levels in vivax malaria strongly correlate with a drop in platelet counts, a data confirmed in two different hospitals of the Amazon area. Although it is not possible, at this point, to speculate on the role of platelets in the increase of CNAs levels in plasma, our results indicate that CNAs might contribute to cell activation and inflammation that are associated with malaria infection.

Although *P. falciparum* infection was not the main scope of the present study, by having access to a small group of patients, it was possible to demonstrate that CNAS levels are increased during acute *P. falciparum* infection. In this malaria model, increased CNAs levels in plasma were associated with thrombocytopenia and the occurrence of fever at the time of blood collection (Figure S3). While these results support the association between CNAS and malaria, the size of our sample precludes any definitive comparison between *P. falciparum* and *P. vivax* infection. Further studies will be required to proper address this question.

In uncomplicated *P. vivax* malaria, we have recently shown that the levels of circulating platelet-derived microparticles (PMPs) are associated with the clinical spectrum of disease, including fever and prolonged time with malaria symptoms [10]. The fact that CNAs levels as well as PMPs were higher in febrile and symptomatic vivax patients suggests a possible association with PMP and CNAs. MPs are important carriers of membrane components or bioactive molecules and their association with nucleic acids has been proposed [49]. The presence of host and/or parasite DNA associated with MPs circulating in plasma and their role in inflammation is currently being addressed in your laboratory.

To investigate if parasite derived-sequences are part of the pool of nucleic acids circulating in blood during vivax malaria, and if these sequences correlate with disease morbidity, we assessed the levels of a parasite specific single copy genomic sequence in CNAs purified from *P. vivax* patients. Although our results revealed that host and parasite sequences are part of the total plasma CNAs levels in acute *P. vivax* infected patients, the levels of a host specific (hTERT) but not parasite specific sequence correlated with vivax clinical disease. These results are in agreement with a recently study carried out in the Amazon area in which high parasitemia was not the rule among patients with severe disease according to the WHO criteria [50].

Whether CNAs are merely inert debris of cellular injury, or if they possess pro-inflammatory properties and are, therefore, players in the immunopathogenic basis of malaria requires further investigation. Although at this point is not possible to draw conclusions, their role in the inflammatory response during malaria cannot be rule out. In fact, it is well known that dying cells spill their content and release a myriad of endogenous pro-inflammatory danger signals, including proteins, nucleic acids, extracellular matrix components, lipid mediators and adenosine triphosphate (ATP) [51]. These endogenous danger signals have been shown to play important roles in inflammation [51], [52], [53]. As human and parasite derived nucleic acid sequences have been shown to posses immune-stimulatory properties, the implication of CNAs in cellular activation and in innate immunity is likely. Likewise, the frequency of immune stimulatory vs. non-stimulatory circulating nucleic acids in plasma from patients with different clinical outcomes would provide important insights into the role of CNAS in malaria pathogenesis.

In conclusion, we show that host circulating nucleic acids in plasma constitute a reliable and non-invasive biomarker to evaluate vivax malaria morbidity. CNAs levels were closely associated with *P. vivax* malaria clinical spectrum, and may have a role in malaria-induced inflammation. Given the enormous economic scourge of *P. vivax* in endemic areas, plasma CNAs levels provide a welcome prognostic tool to rapidly identify potentially severe cases and improve clinical management.

## Materials and Methods

### Study area and subjects

This study was conducted in May 2010, at Fundação de Medicina Tropical Dr. Heitor Vieira Dourado (FMT-HVD), a tertiary care center for infectious diseases in Manaus (3°8′S, 60°1′W), the capital of the state of Amazonas, Brazil. Manaus is clearly part of a new frontier in the economic development of the Amazon and is considered as one of the leading cities in terms of number of *P. vivax* malaria cases in Latin America [54]. In 2009, a total of 19,698 malaria cases were reported in Manaus with a large dominance of vivax (92.6%) over falciparum malaria [55].

Individuals who sought care at FTM-HVD and whose thick blood smear was positive for *P. vivax* were invited to participate in the study. Exclusion criteria included: (i) refuse or inability to sign the informed consent; (ii) age <18 years; (ii) pregnant women; (ii) mixed infection with *P. falciparum* or *P. malariae*; (iv) any other co-morbidity that could be traced. Twenty-one patients, aging 21 to 72 years, were enrolled in the study. Selected volunteers were all negative for *P. falciparum* and/or *Plasmodium malariae* infection by both microscopic examination and a nested-PCR, carried out latter in our laboratory. Clinical and demographical data were acquired through a standardized questionnaire, and the hematological profiles were assessed by automated complete blood count carried out at FMTA hematology facility. Table 1 summarizes demographic, epidemiological, parasitological and hematological data of *P. vivax* infected-volunteers.

**Table 1:** Characteristics of the *Plasmodium vivax* patients enrolled in the study.

CHARACTERISTICS	
***Demographical and epidemiological***	
Sex, male/female, proportion	13/8
Age, median, range	49 (21–72)
N° of previous malaria episodes	3 (0–30)
***Parasitological and hematological*** **, median (range)**	
Parasitemia, parasites/µl of blood	305 (25–2255)
Hematocrit %,	42.6 (30.5–48.9)
Hemoglobin levels g/dL	13.2 (9.5–14.9)
WBC counts×10^6^/mm^3^	4.9 (2–8.6)
RBC counts×10^6^/mm^3^	4.8 (3.57–5.42)
Platelet counts×10^6^/mm^3^	125.5 (39–225)
MCV (fL)	89.3 (82.9–96)
MPV (fL)	9.8 (8.1–13.2)
MCH (pg)	27.1 (25–30.1)
MCHC (g/dL)	30.6 (29.8–33.2)
***Clinical parameters***	
Duration of symptoms in days, median, (range)	3 (>1–20)
Fever at the time of blood sampling, n (%)	7 (33.3%)
***Symptoms in the last 3 days, n (%)***	
Fever	21 (100%)
Myalgia	21 (100%)
Chills	19 (90.5%)
Headache	18 (85.7%)
Nausea	15 (71.4%)
Anorexia	12 (57.1%)
Vomiting	6 (28.6%)
Dyspnea	6 (28.6%)
Diarrhea	3 (14.3%)

The study was approved by the Ethical Review Board of the René Rachou Research Center, FIOCRUZ, Brazilian Ministry of Health (Reporter CEPSH/CPqRR 05/2008). All participants were instructed about the objectives of the study and signed an informed consent in accordance with guidelines for human research, as specified by the Brazilian National Council of Health (Resolution 196/96). Patients diagnosed with vivax malaria were treated according to the standard protocols recommended by the National Malaria Control Program (chloroquine plus primaquine).

Peripheral blood samples (10 mL in EDTA) were obtained from patients on admission and, in those who attended follow-up, during convalescence 7 days later. Plasma samples from 14 age-matched malaria-unexposed donors from Belo Horizonte, a malaria free area, were used as baseline control. Aiming to avoid bias of selection, we further include an additional group of *P. vivax* patients (n = 14; age range, 18–41 years) from a second hospital of the Amazon area, Julio Muller Hospital, Cuiaba, MT, which was located about 1500 miles from Manaus city. CNAS levels were also evaluated in plasma samples from a small group of *P. falciparum* patients (n = 9; age range, 18–52 yrs.). Plasma samples were isolated immediately after blood sampling and stored at −80°C until use.

### Malaria vivax clinical score

Since at present no clear criteria define vivax malaria severity, the present study used the World Health Organization standard criteria built for *P. falciparum* malaria [50]. One patient (Pv_04, Table 1) presented clinical signs of severe malaria according to the WHO criteria. This patient presented with hyperbilirrubinemia (total bilirubin = 4.3 mg/dL) and acute renal failure (creatinin = 2.3 mg/dL), and other common infectious diseases were ruled out during his hospitalization. To define different degrees of morbidity for the remaining *P. vivax* malaria patients, we adapted the criteria originally described by Karunaweera et al [56], and previously validated in the Amazon area [57]. Briefly, the occurrence of fever at the time of blood collection and other 8 signs and/or symptoms that commonly accompany a malarial infection - headache, chills, myalgia, nausea, vomiting and diarrhea - were addressed into the questionnaire applied to each patient. Additionally, hematological parameters were also included in the score calculation: white blood cells (WBC), red blood cells (RBC) and platelets counts, hemoglobin and hematocrit levels (Table 1). Numerical scores of 0 or 1 were assigned to clinical and hematological parameters reported as absent (or within normal range) or present (or outside normal range), respectively. For those 15 parameters analyzed, the sum of scores provides the patient's final clinical score, as shown in Table S1 (supporting information). This semi quantitative clinical assessment enabled numerical comparisons between the plasma CNAs levels and the clinical spectrum of vivax malaria.

### Purification and quantification of CNAs from plasma

Cell-free circulating nucleic acids (CNAs) were isolated from plasma from *P. vivax* patients or healthy donors with QIAamp Circulating Nucleic Acid Kit (Qiagen, CA, US) according to the manufacturer's instructions. Two different methodologies were used to quantify CNAs levels in plasma: (i) amplification of the genomic sequence of the human telomerase reverse transcriptase (hTERT), an ubiquitous single copy gene mapped on 5p 15.33, used here as a marker of the total amount of DNA present in plasma samples. For that, we used the following specific primers Fw: 5′GGC ACA CGT GGC TTT TCG 3′; Rev: 5′ GGT GAA CCT GCT AAG TTT ATG CAA 3′, previously described [58]. To normalize the amount of DNA in plasma samples, 5 ng of Salmon Sperm DNA solution (Invitrogen, CA, USA) were spiked into plasma samples before purification of CNAs. The genomic sequence of the chum salmon (*Oncorhynchus keta*) Y-chromosome specific marker was amplified in parallel with hTERT using the specific primers: Fw: 5′ AGG CAA CCC TTG CTC GAA TT 3′; Rev 5′ TGG GCA CAT GGC TTA CCG 3′; (ii) total dsDNA levels in plasmas were also quantified fluorometrically using the Quant-iTTM Pico Green Reagent (Molecular Probes, Netherlands) according to the manufacturer's instructions.

To identify parasite derived sequences in plasma samples from infected patients the following primer pair Fw: 5′ CAA CAG GTC CTT CAC GCT TAG TG 3′; Rev: 5′ CGA CAG CAC CAT TGG CG 3′ was designed based on the *P. vivax* genomic sequence [59] retrieved from PlasmoDB version 6.4 (http://plasmodb.org/plasmo/). The Primer Express software (PE Applied Biosystems) was used for primer design. Quantitative PCR reactions were carried out in an ABI Prism 7000 Sequence Detection System SDS (PE Applied Biosystems, CA, USA). The temperature profile was 95°C for 10 min followed by 40 cycles of denaturation at 95°C for 15 s and annealing/extension at 60°C for 1 min. The cycle threshold for DNA quantification was set to 0.2 for all experiments in this study.

### Statistical analysis

Data were analyzed using GraphPad Prism version 5.00 for Windows (GraphPad Software, CA, US). Differences in the means were analyzed using two-tailed student's t test or Mann-Whitney test when data did not fit a Gaussian distribution. Spearman nonparametric correlation coefficient was used to analyze the association between the variables.

## Supporting Information

Figure S1 **Absolute quantification of hTERT levels in plasma from P. vivax patients.** The human genomic sequence of hTERT was amplified by PCR using the primers described in M&M. The concentration of the PCR product was determined spectrophotometrically using Nanodrop. (A) A standard curve was built by re-amplifying known amounts of the hTERT PCR product in 10-fold serial dilutions. (B) Amplification of hTERT in CNAs samples purified from healthy donors or malaria patients. (C) Results of interpolated hTERT concentrations in CNAs samples purified from plasma of healthy donors or malaria patients. Levels are expressed as pg/ml. Differences were calculated by the Mann-Whitney test. A p value<0.05 was considered significant.
(TIFF)

Figure S2 **Plasma CNAs levels correlates with vivax thrombocytopenia in a different Brazilian endemic area, Cuiaba, Mato Grosso.** Correlation of plasma CNAs levels with platelet counts in 14 symptomatic vivax malaria patients attended at the hospital Julio Muller, Cuiaba, MT. (A) The mean cycle threshold for hTERT amplification was plotted against the platelet counts (Pearson r = 0.745, *p = 0.0072*). (B) Assessment of CNAs levels and mean cycle threshold for hTERT amplification in samples from 10 out of 14 patients who returned after 7–10 days post treatment.
(TIFF)

Figure S3 **Plasma CNAs levels correlates with thrombocytopenia in** ***P. falciparum*** **patients.** CNAs levels were assessed in plasma from 9 samples from *P. falciparum* patients and correlated with (A) their platelet counts and (B) body temperature measured at the time of blood collection. Fluorometric dsDNA measurement by PicoGreen and qPCR amplification of hTERT genomic sequence were used for comparisons.
(TIFF)

Table S1 **Patient final clinical score and plasma CNAs levels.**
(DOC)
