# Supplementary figures and images for: Plasma Circulating Nucleic Acids Levels Increase According to the Morbidity of Plasmodium vivax Malaria
Source: PLoS One. 2011 May 17;6(5):e19842. doi: 10.1371/journal.pone.0019842 (PMC3096648; doi:10.1371/journal.pone.0019842)

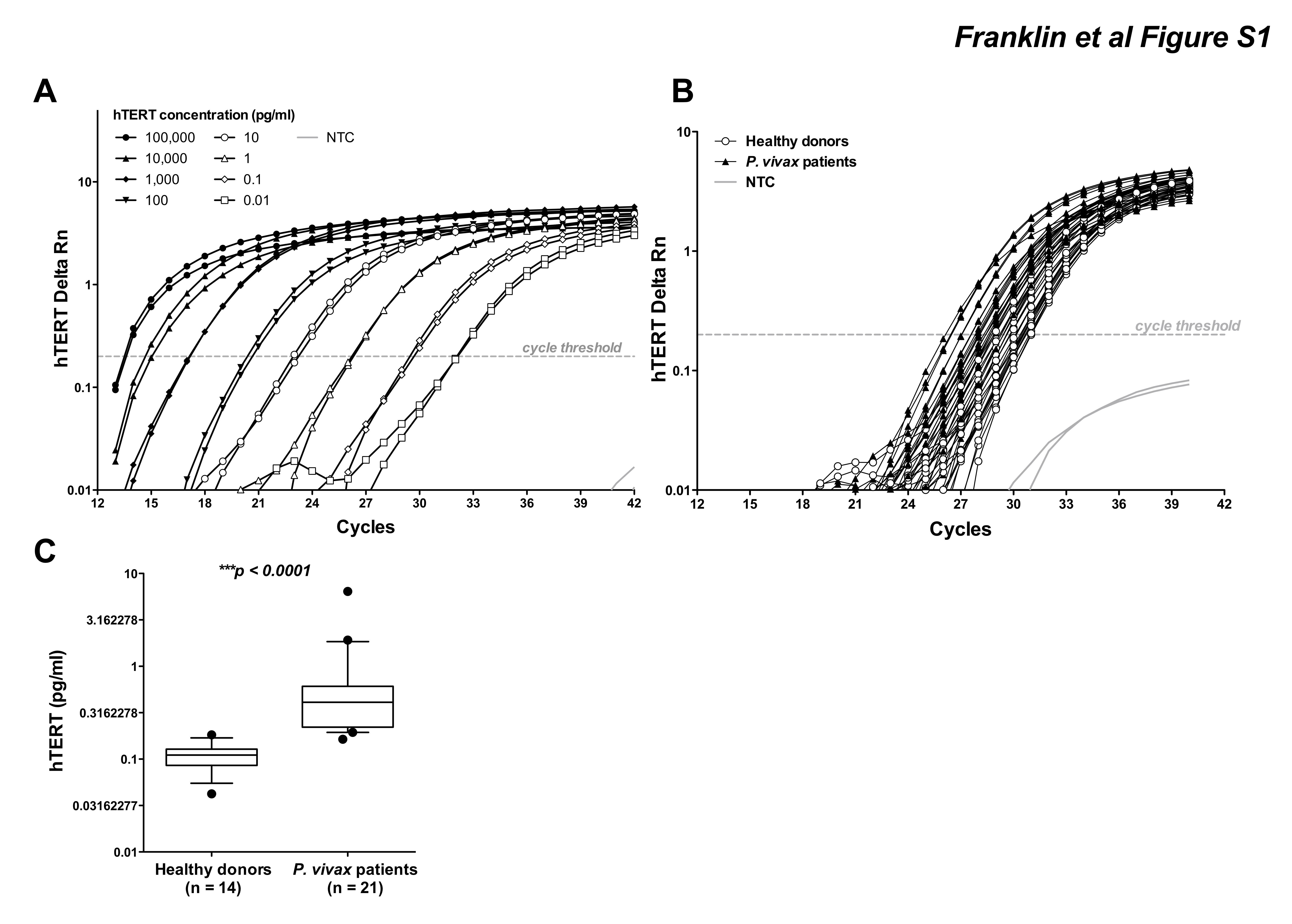

Supplement: Figure S1 — Absolute quantification of hTERT levels in plasma from P. vivax patients. The human genomic sequence of hTERT was amplified by PCR using the primers described in M&M. The concentration of the PCR product was determined spectrophotometrically using Nanodrop. (A) A standard curve was built by re-amplifying known amounts of the hTERT PCR product in 10-fold serial dilutions. (B) Amplification of hTERT in CNAs samples purified from healthy donors or malaria patients. (C) Results of interpolated hTERT concentrations in CNAs samples purified from plasma of healthy donors or malaria patients. Levels are expressed as pg/ml. Differences were calculated by the Mann-Whitney test. A p value<0.05 was considered significant. (TIFF) [file pone.0019842.s001.tiff]

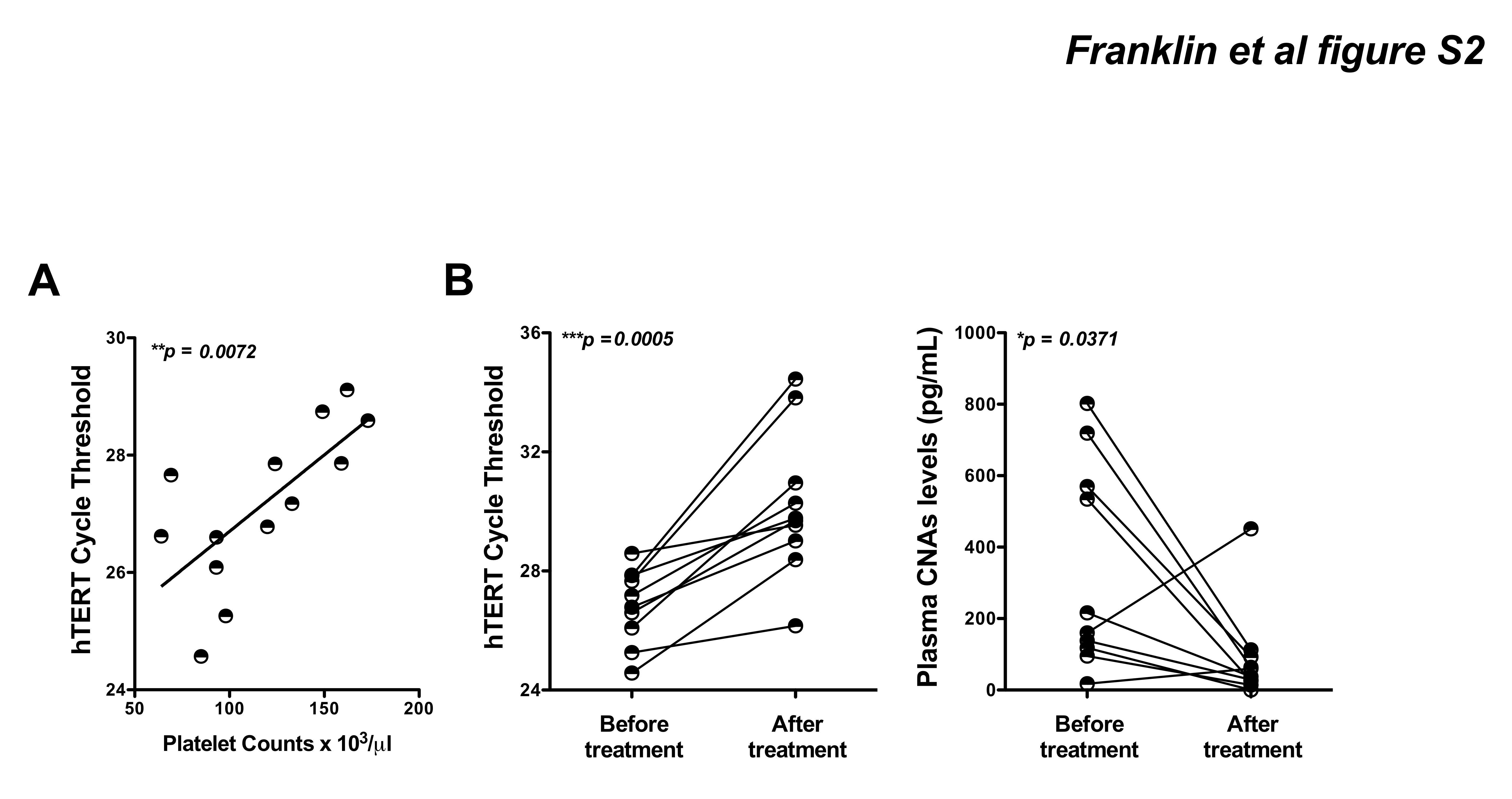

Supplement: Figure S2 — Plasma CNAs levels correlates with vivax thrombocytopenia in a different Brazilian endemic area, Cuiaba, Mato Grosso. Correlation of plasma CNAs levels with platelet counts in 14 symptomatic vivax malaria patients attended at the hospital Julio Muller, Cuiaba, MT. (A) The mean cycle threshold for hTERT amplification was plotted against the platelet counts (Pearson r = 0.745, p = 0.0072). (B) Assessment of CNAs levels and mean cycle threshold for hTERT amplification in samples from 10 out of 14 patients who returned after 7–10 days post treatment. (TIFF) [file pone.0019842.s002.tiff]

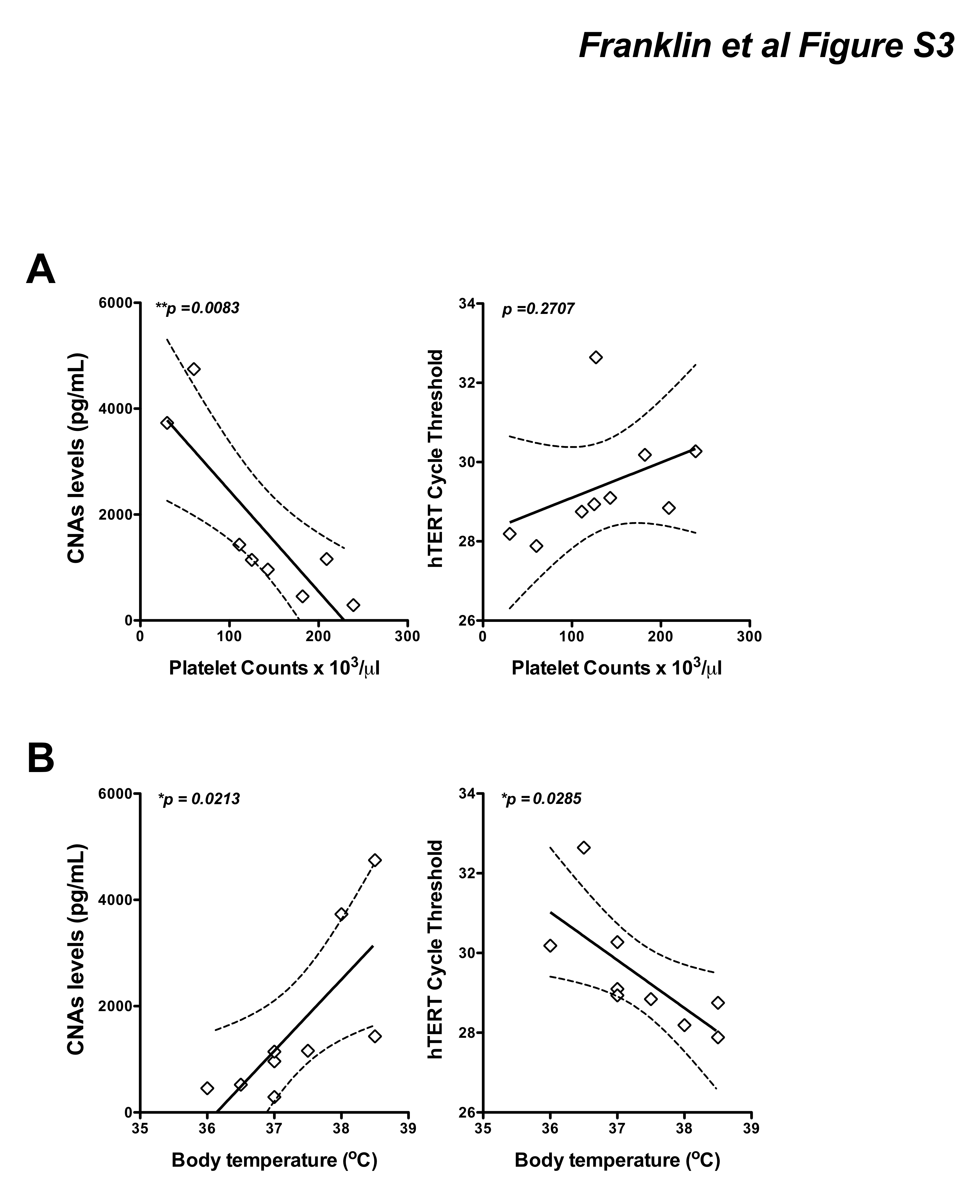

Supplement: Figure S3 — Plasma CNAs levels correlates with thrombocytopenia in P. falciparum patients. CNAs levels were assessed in plasma from 9 samples from P. falciparum patients and correlated with (A) their platelet counts and (B) body temperature measured at the time of blood collection. Fluorometric dsDNA measurement by PicoGreen and qPCR amplification of hTERT genomic sequence were used for comparisons. (TIFF) [file pone.0019842.s003.tiff]
