# Supplementary material for: Plasma Circulating Nucleic Acids Levels Increase According to the Morbidity of Plasmodium vivax Malaria
Source: PLoS One. 2011 May 17;6(5):e19842. doi: 10.1371/journal.pone.0019842 (PMC3096648; doi:10.1371/journal.pone.0019842)
Supplement: Table S1 — Patient final clinical score and plasma CNAs levels. (DOC) [file pone.0019842.s004.doc]

**Supporting Table S1 - Patient final clinical score and plasma CNAs levels**

| **Patient ID** | **Clinical Score** | **CNAs level (ng/ml)** | ***Mean hTERT Ct** |
| --- | --- | --- | --- |
| Pv_01 | **5** | **853.3** | **29.3** |
| Pv_02 | **5** | **1165.3** | **28.9** |
| Pv_03 | **11** | **1991** | **26.3** |
| Pv_04 | **13** | **5843** | **24.7** |
| Pv_05 | **6** | **1083.4** | **27.3** |
| Pv_06 | **7** | **2780** | **26.8** |
| Pv_07 | **5** | **800.5** | **29.1** |
| Pv_08 | **4** | **933.5** | ND |
| Pv_09 | **9** | **1929.2** | **28.2** |
| Pv_10 | **6** | **694.3** | **29.1** |
| Pv_11 | **7** | **1821.7** | **27.1** |
| Pv_12 | **4** | **753.7** | **28.6** |
| Pv_13 | **4** | ND | **29.1** |
| Pv_14 | **4** | **1131.1** | **28.6** |
| Pv_15 | **8** | **1019.1** | **28.4** |
| Pv_16 | **4** | **960.1** | **28.2** |
| Pv_17 | **7** | **817.2** | **28.6** |
| Pv_18 | **6** | **1019.5** | **29.3** |
| Pv_19 | **7** | **1181.7** | **27.6** |
| Pv_20 | **6** | **933.1** | **28.1** |
| Pv_21 | **7** | **2183.5** | **27.4** |

* Cycle threshold obtained after qPCR amplification of the human genome sequence hTERT (means of quadruplicates).
